# Maternal Expression Relaxes Constraint on Innovation of the Anterior Determinant, *bicoid*

**DOI:** 10.1371/journal.pgen.0010057

**Published:** 2005-11-11

**Authors:** Michael S Barker, Jeffery P Demuth, Michael J Wade

**Affiliations:** Department of Biology, Indiana University, Bloomington, Indiana, United States of America; Princeton University, United States of America

## Abstract

The origin of evolutionary novelty is believed to involve both positive selection and relaxed developmental constraint. In flies, the redesign of anterior patterning during embryogenesis is a major developmental innovation and the rapidly evolving *Hox* gene, *bicoid (bcd),* plays a critical role. We report evidence for relaxation of selective constraint acting on *bicoid* as a result of its maternal pattern of gene expression. Evolutionary theory predicts 2-fold greater sequence diversity for maternal effect genes than for zygotically expressed genes, because natural selection is only half as effective acting on autosomal genes expressed in one sex as it is on genes expressed in both sexes. We sample an individual from ten populations of *Drosophila melanogaster* and nine populations of *D. simulans* for polymorphism in the tandem gene duplicates *bcd,* which is maternally expressed, and *zerknüllt (zen),* which is zygotically expressed. In both species, we find the ratio of *bcd* to *zen* nucleotide diversity to be two or more in the coding regions but one in the noncoding regions, providing the first quantitative support for the theoretical prediction of relaxed selective constraint on maternal-effect genes resulting from sex-limited expression. Our results suggest that the accelerated rate of evolution observed for *bcd* is owing, at least partly, to variation generated by relaxed selective constraint.

## Synopsis

How do novel structures and functions originate? This question has proven more difficult to answer than the question of how existing structures are refined to better suit the environment. Evolution by natural selection explains the latter. Ironically, it is the power of natural selection to maintain genes that are suitable for their current roles that also works against the evolution of entirely new traits. The conservation of genes controlling the early stages of embryo development, from worms, to flies, to humans, is a famous example of natural selection's power to constrain evolution and thwart the spread of evolutionary novelties. However, the gene determining which end of the fly embryo becomes the head has a relatively recent origin. How did this gene, *bicoid,* escape purifying selection and take on a novel function? The authors investigate the hypothesis that because *bicoid* is expressed only in females it experiences only half as much constraint as a gene expressed in both sexes. Comparing sequences of *bicoid* with its duplicate gene *zerknüllt,* which retains expression in both sexes, the authors show that, as expected, the variation in *bicoid* is twice that of *zerknüllt*. The findings suggest that relaxed constraint is an important step in the origin of novel function.

## Introduction

The origin of novel structures and functions has long been problematic for evolutionary biology. A century-long debate has centered on the relative contribution of deterministic processes, such as natural selection, and stochastic processes, such as mutation and random genetic drift [1,2] to biological innovation. In modern parlance, “There are problems specific for the origin of novelties that are not the same as in the case of adaptive modification of existing structures ... novelties apparently arise in spite of strong developmental constraints that generally canalize morphological evolution” [3]. How descendant lineages overcome ancestral constraint is a principal research objective in the study of evolutionary novelty.

One route to gaining novel structures and functions is by relaxing ancestral evolutionary constraints. Although the degree of evolutionary lability can be discerned from the phylogenetic distribution of a given trait, the inference of relaxed constraint is more difficult and potentially tautological [4,5]. We show how to test the hypothesis of relaxed constraint using population genetic theory and sequence data from the novel *Hox* gene, *bicoid (bcd),* a critical component of the redesign of anterior patterning during fly embryogenesis, a major developmental innovation [6,7].

The gene *bcd* is present only in the derived Cyclorrhaphan flies, and is the result of duplication and divergence of a more widely conserved *Hox3* descendant *zerknüllt (zen)* [7,8]. After the origin of myriapods in the arthropod lineage leading to insects, *Hox3* underwent a functional transformation to what is now called *zen* [9,10]. In taxa prior to the duplication giving rise to *bcd, zen* is expressed both maternally and zygotically and is important for early designation of both embryonic and extraembryonic tissues [11]. Following duplication, the ancestral maternal-zygotic *zen* expression pattern was partitioned into solely maternally expressed *bcd* and solely zygotic expressed *zen* [6,8]. Since the partitioning of gene expression, *bcd* has evolved faster than *zen* [12,13].

The preservation of both gene copies and the partitioning of the ancestral maternal-zygotic expression pattern between them suggest that functional constraints on both genes remain despite *bcd* evolving additional, unique functionality. Furthermore, because both are homeotic genes critical to early development, selection on the coding regions of these genes is likely to be predominantly purifying (i.e., the selection coefficient, *s,* has tended to be negative). Thus, we can reasonably assume that the effective population size, *N*_e_, the regional chromosomal environment (affecting mutation and recombination), and the average magnitude and sign of *s* have been approximately the same for both genes. There are several factors that might contribute to violations of our assumption of equivalent *s*. However, it is our purpose to test whether the reduced efficacy of selection that attends sex-limited expression is sufficient to explain the pattern of *bcd* diversity without invoking changes to *s*.

From a population genetics perspective, evolutionary constraint can be relaxed by either reducing the strength of purifying selection or limiting the proportion of genes exposed to selection. Here we refer to the former as relaxed selection and the latter as relaxed constraint. Population genetic theory shows that deleterious alleles at autosomal loci with sex-limited effects have equilibrium frequencies twice as high as those expressed in both sexes with similar fitness effects [14,15]. This relaxed constraint is due to deleterious alleles being “hidden” from selection in half of the population (i.e., males, assuming 1:1 sex ratio) in much the same way that recessive alleles have higher expected equilibrium frequencies (i.e., relaxed constraint) because they are “masked” from selection in heterozygotes. If *bcd* is experiencing relaxed constraint because it has sex-limited expression, then we expect the coding regions of *bcd* to be twice as diverse as those of *zen* [13]. Here, we report the first, to our knowledge, test of the prediction that maternal-effect genes have a 2-fold higher heterozygosity at mutation-selection balance than zygotically expressed genes by comparing within species sequence diversity for *bcd* and *zen* from ten populations of *Drosophila melanogaster* and nine of *D. simulans*.

## Results

At mutation-drift balance, the expected value of Tajima's *D* is near zero [16,17] with positive (balancing selection) or negative (purifying selection) values caused by selection. The largest deviation from zero among our estimates of Tajima's *D* is −1.01513 for *D. melanogaster bcd,* and the smallest is −0.42264 for *D. melanogaster zen*. None of the values are significantly different from zero (or from one another), suggesting both genes in both species are near equilibrium (Table 1).

**Table 1:**
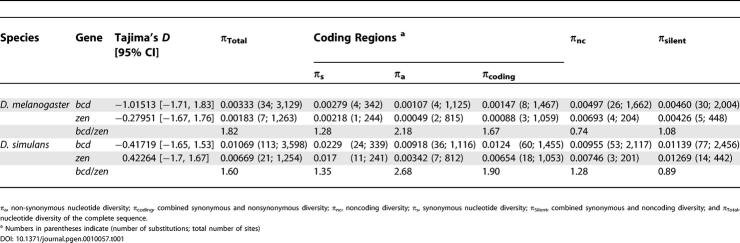
Tajima's *D* and Nucleotide Diversities (π) for *bcd* and *zen*

The observed *bcd*/*zen* ratios of total sequence diversity are 1.82 for *D. melanogaster* and 1.60 for *D. simulans*. For the noncoding regions, the ratios are 0.74 and 1.28, respectively, while coding diversity ratios are 1.67 and 1.90 (Table 1). Further partitioning the diversity in coding regions, the *bcd*/*zen* diversity ratios for synonymous sites are 1.28 (0.00279/0.00218) for *D. melanogaster* and 1.35 (0.0229/0.017) for *D. simulans*. For the nonsynonymous sites in coding regions, the ratios are 2.18 (0.00107/0.00049) and 2.68 (0.00918/0.00342), respectively. Combining the nucleotide diversity of noncoding and synonymous sites for each gene in each species we find the *bcd*/*zen* ratios at silent sites to be 1.08 for *D. melanogaster* and 0.89 for *D. simulans,* very close to the theoretical expectation of 1.0. In contrast, for nonsynonymous sites in the coding regions, the ratios are somewhat in excess of 2, the theoretically predicted value.

## Discussion

The redesign of anterior patterning during embryogenesis is a major developmental innovation, involving both positive selection and the overcoming of developmental constraints. A previous study of sequence variation in *bcd* from a Zimbabwe population of *D. melanogaster* [18] found a significant excess of replacement polymorphisms, indicative of reduced purifying selection, as well as “extensive” haplotype structure consistent with positive selection and linkage disequilibrium. The authors concluded that the selective history of *bcd* is “Complex, involving both relaxation of purifying selection in some parts of the protein and the action of selection in other parts of the gene region” [18]. We do not find evidence of haplotype structure; however, this absence is likely the result of sampling single flies from geographically diverse populations instead of the more intensive sampling of a single population, as in the study of Zimbabwe *D. melanogaster*.

Our comparison of *bcd* and *zen* sequence diversity suggests that the variation necessary for *bcd* to overcome developmental constraint is not due solely to redundancy attending gene duplication [13], but is more consistent with the relaxed constraint that accompanies sex-limited expression. Furthermore, our results indicate that most of the difference in *bcd* and *zen* variation can be accounted for by relaxed constraint without invoking differences in *s*. Although we find no extensive haplotype structure in our analysis, its existence in prior studies may be due to occasional selective sweeps that fix the high standing diversity of *bcd* within populations.

In the origin of evolutionary novelty, the balance between positive selection and relaxed constraint may be different for maternally expressed genes than for paternally expressed genes [19,20]. Although similarly expressed in only half of the members of a population, sexual selection acting on males through paternally expressed genes can be much stronger than the viability selection acting on embryos as a result of maternal-effect genes [21]. Maternal-effect genes are more similar in their evolutionary genetics to aging genes expressed late in life. Alleles with detrimental effects late in life accumulate in a population because selection against them is weaker than it is for alleles with deleterious effects of similar magnitude expressed earlier [22,23]. However, it is difficult to make a quantitative prediction regarding the elevated heterozygosity of aging genes because the degree of relaxed selective constraint depends upon a host of demographic variables. For maternal effect genes, we expect a 2-fold increase in heterozygosity at mutation-selection balance, because they are “hidden” from natural selection in half the population, i.e., in males, where they are not expressed. The levels of *bcd* and *zen* sequence diversity we observed in *D. melanogaster* and *D. simulans* support this theoretical prediction.

## Materials and Methods

We purchased ten *D. melanogaster* and nine *D. simulans* wild-type strains from the Tucson *Drosophila* Species Stock Center (http://stockcenter.arl.arizona.edu/) (Table S1). We selected strains from a broad geographic range to sample genetic variation across the distribution of each species. From each strain, a single individual was randomly selected for DNA extraction and analyses of *bcd* and *zen* sequence diversity.

Genomic DNA was extracted from single flies using the Puregene tissue kit (Gentra Systems, Minneapolis, Minnesota, United States). Oligonucleotides for PCR amplification and direct sequencing were designed using the FastPCR program [24] from previously published *D. melanogaster* and *D. simulans bcd* and *zen* sequences. To increase primer specificity and avoid amplifying other genes, candidate primers were searched against the *D. melanogaster* genome to remove primers that may amplify other genes. Further, in silico PCR simulations of candidate primer pairs against all members of the Antennapedia Complex were conducted in FastPCR [24] to remove primers that may amplify related sequences.

The *bcd* primer pairs amplified a ~4-kb region that included ~60 bp of the 5′ and ~1 kb of the 3′ flanking regions. The *zen* primer pairs amplified an ~1.3-kb region that included ~30 bp of the 5′ and ~100 bp of the 3′ flanking regions. PCR products were purified using the QIAquick PCR Purification Kit (Qiagen, Valencia, California, United States). Forward and reverse strands were sequenced on an ABI 3730 DNA Analyzer (Applied Biosystems, Foster City, California, United States) using Big Dye Terminator v 3.1 chemistry (Applied Biosystems). Oligonucleotides used for PCR amplifications were also used for sequencing the *D. melanogaster* and *D. simulans bcd* and *zen* genes. The resulting sequences are deposited in GenBank (http://www.ncbi.nlm.nih.gov/) with the following accession numbers: *D. melanogaster bcd* (DQ114428–DQ114437), *D. melanogaster zen* (DQ114438–DQ114447), *D. simulans bcd* (DQ114448–DQ114456), and *D. simulans zen* (DQ114457–DQ114465).

We constructed contigs for each gene using CodonCode Aligner v. 1.3.4 (CodonCode, Dedham, Massachusetts, United States). All base calls in each contig were based upon at least one forward and reverse sequence pair, with most bases covered by two forward and reverse pairs. Contigs of each gene were aligned using BioEdit v. 7.0.4.1 [25]. We verified each alignment and variable site manually. Coding regions were defined for *bcd* following Baines et al. [18]; i.e., only the major transcript was considered coding. For our *bcd* sequences, we defined the coding regions of *D. melanogaster* as positions 68–232, 792–867, 923–1,864, and 2,377–2,663. For our *D. simulans bcd,* the coding regions were defined as positions 68–232, 776–851, 907–1,848, and 2,235–2,521. Coding regions for the *zen* sequences were identified by comparison to the *D. melanogaster* reference sequence. In our *D. melanogaster zen* sequences, the coding region was defined as positions 27–113 and 178–1,152. For *D. simulans zen*, the coding region was defined as positions 27–113 and 178–1,146.

Nucleotide diversity, π, is the average number of nucleotide differences per site between two randomly chosen sequences. It was calculated according to Nei [26] using DnaSP 4.10 [27]. To assess neutrality, we estimated Tajima's *D* [17]. Then, using the observed Tajima's *D* and the average number of nucleotide differences per sequence, we computed 95% confidence intervals by coalescent simulation in DnaSP.

## Supporting Information

Table S1 Stock Center Accession Numbers and Collection Locations of *Drosophila* Populations (41 KB DOC)

### Accession Numbers

The GenBank (http://www.ncbi.nlm.nih.gov/) accession numbers discussed in this paper are *D. melanogaster bcd* (AF466621–AF466645), *D. simulans bcd* (AF465792), and *D. melanogaster zen* reference sequence (NT_033777). *D. melanogaster* GeneIDs are *bcd* (40830) and *zen* (40828).
