# Supplementary material for: Maternal Expression Relaxes Constraint on Innovation of the Anterior Determinant, bicoid
Source: PLoS Genet. 2005 Nov 11;1(5):e57. doi: 10.1371/journal.pgen.0010057 (PMC1283158; doi:10.1371/journal.pgen.0010057)
Supplement: Supplementary file 1 [file pgen.0010057.st001.doc]

**Supporting Information**

**Table S1. Stock center accession numbers and collection locations of *Drosophila* populations.**

| Tucson Stock Center Accession # | Species | Collection Location |
| --- | --- | --- |
| 231.00 | *D. melanogaster* | Oahu, Hawaii, USA |
| 231.01 | *D. melanogaster* | Ica, Peru |
| 231.03 | *D. melanogaster* | Toonda, Australia |
| 231.04 | *D. melanogaster* | Kuala Lumpur, Malaysia |
| 231.05 | *D. melanogaster* | Agana, Guam |
| 231.06 | *D. melanogaster* | Mysore, India |
| 231.14 | *D. melanogaster* | Orlando, Florida, USA |
| 231.15 | *D. melanogaster* | Bahia, Brazil |
| 231.20 | *D. melanogaster* | Rancho Zapata Minatitlan, Oaxaca, Mexico |
| 231.23 | *D. melanogaster* | Crete, Greece |
| 251.001 | *D. simulans* | Georgetown, Guyana |
| 251.005 | *D. simulans* | Lima, Peru |
| 251.006 | *D. simulans* | Nueva, California, USA |
| 251.007 | *D. simulans* | Rarotango, Cook Islands |
| 251.009 | *D. simulans* | Gorak, New Guinea |
| 251.169 | *D. simulans* | South Africa |
| 251.175 | *D. simulans* | Forster, Australia |
| 251.177 | *D. simulans* | Central Catalina Island, California,USA |
| 251.178 | *D. simulans* | Plum Grove, Chiapas, Mexico |
